# Macro- and Micro-Heterogeneity of Natural and Recombinant IgG Antibodies

**DOI:** 10.3390/antib8010018

**Published:** 2019-02-19

**Authors:** Alain Beck, Hongcheng Liu

**Affiliations:** 1 Biologics CMC and developability, IRPF, Center d’immunologie Pierre Fabre, St Julien-en-Genevois CEDEX, 74160 Saint-Julien en Genevois, France; 2 Anokion, 50 Hampshire Street, Suite 402, Cambridge, MA 02139, USA

**Keywords:** critical quality attributes, comparability, developability, glycosylation, quality target product profile, mass spectrometry, post-translational modifications, proteoforms, safety

## Abstract

Recombinant monoclonal antibodies (mAbs) intended for therapeutic usage are required to be thoroughly characterized, which has promoted an extensive effort towards the understanding of the structures and heterogeneity of this major class of molecules. Batch consistency and comparability are highly relevant to the successful pharmaceutical development of mAbs and related products. Small structural modifications that contribute to molecule variants (or proteoforms) differing in size, charge or hydrophobicity have been identified. These modifications may impact (or not) the stability, pharmacokinetics, and efficacy of mAbs. The presence of the same type of modifications as found in endogenous immunoglobulin G (IgG) can substantially lower the safety risks of mAbs. The knowledge of modifications is also critical to the ranking of critical quality attributes (CQAs) of the drug and define the Quality Target Product Profile (QTPP). This review provides a summary of the current understanding of post-translational and physico-chemical modifications identified in recombinant mAbs and endogenous IgGs at physiological conditions.

## 1. Introduction

Recombinant monoclonal antibodies are heterogeneous due to post-translational modifications (PTMs) and physico-chemical transformations that could occur during their entire life-span. Understanding of the mechanisms and the ways to control the heterogeneity are essential to the successful clinical development of monoclonal antibody (mAb) therapeutics. Based on International Conference on Harmonization (ICH) Q6B, mAb variants can be classified as either “Product-related substances” or “Product-related impurities”. Product-related substances are defined as “Molecular variants of the desired product formed during manufacturer and/or storage which are active and have no deleterious effect on the safety and efficacy of the drug product. These variants possess properties comparable to the desired product and are not considered impurities.” Product-related impurities are defined as “Molecular variants of the desired products (e.g., precursors, certain degradation products arising during manufacture and/or storage) which do not have properties comparable to those of the desired product with respect to activity, efficacy, and safety.” Therefore, mAb variants are required to be thoroughly characterized to determine their chemical nature and impact on stability, activity, efficacy, and safety.

Because process changes are inevitable during process development, optimization and scale-up, a thorough understanding of mAb variants is also critical to demonstrating comparability between batches. The acceptance criteria to establish comparability for product-related impurities are more stringent than that of product-related substances (ICH Q5E). Failure to demonstrate the presence of the same type of modifications at comparable levels in post-change materials may require additional preclinical or clinical studies, due to safety concerns. Furthermore, mAb variants with different modifications might impact long-term stability and, thus, shelf-life, efficacy, and safety.

Therapeutic mAbs have evolved from a murine origin, to chimeric, and humanized or fully human to reduce immunogenicity, based on amino acid sequence homology. Generally, human-like modifications, identified as such by their presence in natural Immunoglobulin Gs (IgGs), pose a lower risk of immunogenicity.

This review focuses on the current understanding of the various types of modifications of mAbs, that can occur during manufacturing, storage, and post-administration in vivo or during clinical trials. Known modifications of human endogenous IgGs are also discussed. An overall comparison between the different modifications found in mAbs versus natural IgGs is presented in Table 1.

## 2. N-Terminal Modifications

N-terminal pyroglutamate (pyroGlu) is a common mAb modification resulting mainly from a non-enzymatic cyclization of N-terminal glutamine (Gln) [1,2,3,4,5]. At a much lower rate, N-terminal glutamate (Glu) can also be converted to pyroGlu [6,7,8]. Various environmental factors, such as buffer composition, pH, and temperature during cell culture and purification, can impact the conversion rates, which accounts for the varied levels of N-terminal pyroGlu found in mAbs [1,2,3,4,5]. Conversion of Glu to pyroGlu does not contribute as extensively to N-terminal heterogeneity as does the more commonly observed Gln to pyroGlu conversion because of the dramatic difference in the conversion rates. Cyclization of N-terminal Gln or Glu to pyroGlu reduces the molecular weight of a mAb by 17 Da or 18 Da, respectively. MAbs with the original Gln are more basic than those with pyroGlu [1,2,9], though, the presence of N-terminal pyroGlu has no impact on mAb structure and function [2,8]. The same conversion from Gln to pyroGlu is expected for mAbs in circulation because of the non-enzymatic nature of this reaction. N-terminal Glu has also been shown to be converted to pyroGlu in circulation [6].

Another common N-terminal modification is the incomplete removal of light chain or heavy chain signal peptides, which results in mAbs with truncated signal peptides of varying sizes [2,9,10,11,12,13,14]. The presence of signal peptides with different number and type of amino acids adds mass heterogeneity to mAbs. Interestingly, mAbs with signal peptides have been detected mainly as basic species [2,9,12,13,15], but rarely as acidic species [16]. The presence of low levels of signal peptides has no impact on potency [2,9,13] and pharmacokinetics (PK) in rats [13].

Although it is less common, N-terminal truncation has been reported. A combination of a murine signal peptide and antibody lambda light chain causes an alternative cleavage of the signal peptide resulting in a mAb with the loss of three amino acids from the light chain [17]. With the use of a specific tag to label N-terminal primary amine in combination with liquid chromatography mass spectrometry (LC-MS), an mAb variant with the loss of one amino acid from the light chain was observed [11].

Natural IgG contains approximately 1.8-mole pyroGlu/mole IgG [6]. Based on the different reaction rates, it is expected that most of the pyroGlu originates from N-terminal Gln, rather than Glu. Assuming that the stress condition for massive production of mAbs from ex vivo expression in host cell lines is the cause of N-terminal signal peptides and truncation, they are not expected to occur and have not been reported for natural IgGs.

## 3. Asn Deamidation

Asparagine (Asn) deamidation is almost a ubiquitous modification of mAbs and has been well studied because of its contribution to heterogeneity, and its potential impact on potency and immunogenicity. Asn residues in the complementarity determining regions (CDRs) are inherently susceptible to deamidation because of their relatively higher flexibility and exposure to solvents than at other locations [2,18,19,20,21,22]. Deamidation in CDRs can cause a substantial loss of potency [20,22,23,24]. In addition to deamidation in the CDRs, deamidation also occurs in susceptible Asn residues in the constant region. The most widely observed deamidation site is located in the fragment crystallizable (Fc) region within the amino acid sequence of SNGQPENNY [2,25,26,27,28,29]. Deamidation in the constant regions other than within the commonly observed sequence has also been reported [25,30]. When measured by differential scanning calorimetry, the fragment antigen binding (Fab) fragment with the deamidation product, isoaspartate (isoAsp), is less stable compared to Fab with the original Asn residue [22]. Deamidation increases the molecular weight of mAbs by 1 Da and generates acidic species [2,12,13,20,21,22,31,32]. Variants containing deamidation products are less hydrophobic than those with the original Asn residues [20,32]. Deamidation does not impact in vivo clearance [21,26]. Deamidation of Asn residues continues to occur in vivo in the CDRs [19,21,33,34] and Fc region [26] of mAbs. The in vivo deamidation kinetics can be fully predicted via in vitro stress studies under physiological conditions [19,26], which indicates the same non-enzymatic mechanism. It should be noted that it is important to optimize digestion procedures and distinguish procedure-induced artifact versus real for the accurate determination of Asn deamidation levels.

Natural human IgG has 23% deamidation at the conserved site in the Fc region, which is consistent with the molecules’ in vivo half-life [26]. The presence of high levels of deamidation in natural human IgG suggests that deamidation, at least at the conserved site, is not foreign to the immune system and, therefore, would not present an increased risk of immunogenicity.

## 4. Asp Isomerization

Aspartate (Asp) isomerization has been commonly observed in mAbs in CDRs due to higher levels of flexibility and exposure [15,18,20,35,36,37,38,39,40,41]. Isomerization of Asp in CDRs has been shown to cause a decrease in antigen binding affinity [20,35,36,39,42]. Since there is no charge difference between Asp and isoAsp, the observed decrease in potency is probably caused by conformational changes due to the introduction of a methyl group into the peptide backbone. Isomerization does not change mAb molecular weight; however, depending on the specific location, isomerization can either generate acidic [12] or basic [20,43] species. Similarly, isomerization could result in mAbs or their Fab fragments becoming either more [15,35,37,44] or less [20,45] hydrophobic. Isomerization is a non-enzymatic reaction with an optimal pH of around 5 [23,41]. Under physiological conditions, isomerization was not found to increase for 34 days [26]. Therefore, the level of isomerization is expected to be low in natural IgGs.

## 5. Succinimide

Succinimide is the reaction intermediate of both Asn deamidation and Asp isomerization and is commonly detected in CDRs [15,20,23,34,35,37,41,42,46]. The presence of succinimide in the CDR has been demonstrated to cause a decrease in potency [23,34,35,42]. Succinimide as a deamidation intermediate has also been detected in the conserved susceptible Asn deamidation site [25,28]. MAb variants containing succinimide from Asp isomerization have been shown to become more acidic [43] or basic [15,20,23,41], due to direct charge difference and conformational changes. Similarly, mAb or Fab with succinimide as isomerization intermediate could be more [35,37,44] or less hydrophobic [15]. It is worth mentioning that mAb variants containing succinimide, which alters the molecular weight difference by only 18 Da, have been reported to appear as a back shoulder of the main peak by size exclusion chromatography (SEC) [34,47], suggesting a substantial conformational difference. It has been shown that the succinimide residue contained in mAb was converted to Asp and isoAsp after administration to monkeys [34]. Due to its instability under physiological pH, succinimide is not expected to be detected in natural human IgG. 

## 6. Oxidation

Methionine (Met) residue is the most commonly observed amino acid that is susceptible to oxidation in mAbs. Studies have shown oxidation of Met in the heavy chain CDR2 [39] or the frame work region [48]. Met oxidation did not show a negative impact on antigen binding in either case. Two conserved Met residues close to the heavy chain constant domain 2 (CH2)-CH3 domain interface have been shown to be susceptible to oxidation [48,49,50,51]. The addition of one oxygen atom increases mAb molecular weight by 16 Da. As expected, mAb variants with oxidized Met are less hydrophobic compared to the non-oxidized molecules [44,45,52,53]. Interestingly, one mAb with the Fc conserved Met oxidized appeared to be more basic [51], while, another mAb with an oxidized Met in the Fab region appeared to be more acidic [31]. Oxidation of the two conserved Met residues in the Fc region caused conformational changes mainly in the CH2 domain [49,54] along with a host of negative impacts, including decreased thermal stability, [48,49,50,55] increased aggregation [49,55], decreased complement-dependent cytotoxicity (CDC) [48], decreased binding affinity to neonatal Fc receptor (FcRn) [48,56,57] and shorter in vivo half-life [58].

Oxidation has also been observed at tryptophan (Trp) residues in mAbs [59,60,61,62]. Trp residues in CDRs are more susceptible to oxidation due to a higher level of solvent exposure [63]. Oxidation of Trp generates a number of species, the major ones having molecular weight increases of 16 Da, and 32 Da [62,64]. MAb variants with oxidized Trp are less hydrophobic [52]. Oxidation of Trp residues in the CDRs can lead to reduced potency, decreased thermal stability, and increased aggregation propensity [59,60,61]. Trp oxidation has also been demonstrated to cause yellow coloration of the mAb solution, [64] due to kynurenine formation. 

Oxidation of Met and several other amino acids has been detected in natural human IgG [65,66]. Oxidative stress under various pathological conditions and the resulting reactive oxygen species are expected to cause oxidation of susceptible residues in natural IgGs, as one of the most abundant proteins in circulation. 

## 7. Cysteine and Disulfide Bond

Theoretically, all cysteine residues of mAbs should be involved in the formation of either intra- or inter-chain disulfide bonds in a well-defined linkage pattern. However, several variants that deviate from the well-established IgG disulfide bond structure have been discovered. These variants include the presence of free cysteine (Cys) residues, alternative disulfide bond linkage (scrambling), trisulfide bonding, the formation of thioether, and cysteine racemization.

The presence of free cysteine can be classified into three scenarios. The first scenario is the widely-reported occurrence of free cysteine residues [67,68,69,70,71]. These free cysteines have been shown to lower thermal stability [67] and increase the formation of reducible covalent aggregates [72,73,74]. The second scenario is the detection of relatively high levels of free Cys often due to the incomplete formation of a particular disulfide bond, mainly in the heavy chain variable domain [4,37,44,75,76] or the disulfide bond between the light chain and heavy chain [13,77]. The incomplete variable domain disulfide bond reduces the potency of one mAb [44], but has no impact on a different mAb [75]. MAb variants with the incomplete heavy chain variable domain disulfide bond were separated as acidic species in one case [75], but basic species in the other case [4], indicating that a structural change was likely the cause of different chromatographic behaviors. MAb variants with the incomplete variable domain disulfide bond are more hydrophobic [15,37,44]. MAb variants without the disulfide bond between the light chain and heavy chain are enriched in the acidic species [13,77]. The incomplete heavy chain variable domain disulfide bond can be reformed in vivo [4]. In the third scenario, the mAbs contained an extra non-canonical cysteine residue, mostly in the CDRs. The extra Cys in mAbs can be modified by small thiol containing compounds, such as free cysteines [78,79,80,81], and glutathione, [80,81] or oxidized to form cysteine sulfinic or sulfonic acid [81]. Modification of Cys introduces molecular weight heterogeneity. In addition, mAbs with modified Cys are less hydrophobic compared to unmodified molecules [81]. Cysteinylation increases mAb molecular weight, causes the formation of acidic species, and decreases antigen binding [78]. Modification of the extra Cys residue also causes lower expression titer, decreased thermal stability, and higher propensity towards aggregation [78,80]. 

The alternative disulfide bond linkage was first discovered in IgG4 molecules, where the formation of two inter-heavy chain disulfide bonds is in equilibrium with the formation of two intra-chain disulfide bonds [82,83,84,85]. The direct outcome of this equilibrium is the formation of bispecific antibodies, which has been reported for both recombinant monoclonal antibodies and natural antibodies [82,84]. Mutation of the IgG4 hinge region amino acid sequence, CPSC, to the IgG1 amino acid sequence, CPPC, can eliminate the Fab-exchange phenomena [85,86,87], which has been employed as a strategy to create stable mAb therapeutics based on the IgG4 framework. Later, the alternative disulfide bond linkage in the hinge region of IgG2 antibodies was discovered, both in recombinant and in natural human IgG2 [88]. Different IgG2 isoforms showed a subtle difference in structure and thermal stability [89]. While having no difference in molecular weights, the three disulfide isoforms, A, B, and A/B, can be differentiated using several analytical methods. By ion-exchange chromatography, the B isoform appeared to be more acidic than A/B, followed by A [43,88]. By reversed-phase chromatography, the B isoform eluted from columns earlier than A/B, followed by A [89,90,91]. By capillary electrophoresis sodium dodecyl sulfate (CE-SDS), the A isoform migrated faster than A/B, whereas the B isoform migrated the slowest [88]. Depending on the specific molecule, different isoforms may or may not have an impact on potency [89]. The conversion from A to B through the A/B isoform continues in mAbs in circulation [91]. 

The thioether linkage was first discovered in an IgG1 antibody as a non-reducible species using reducing sodium dodecyl sulfate polyacrylamide gel electrophoresis (SDS-PAGE) and CE-SDS [92] Later, it was found that the thioether between the light and heavy chains can also be formed in mAbs in vivo and in human natural IgGs [93]. The rate of thioether formation in IgG1 containing the lambda chain is faster than the conversion rate in IgG1 containing the kappa chain [93]. The formation of thioether reduces mAb molecular weight by 32 Da due to the loss of a sulfur atom.

Trisulfide bond was first discovered in an IgG2 mAb [94]. It was later found that trisulfide bond occurs in all classes of mAbs and natural human IgGs, mainly between the light chain and heavy chain [93,95,96]. Trisulfide bond formation can be controlled by changing the feeding strategy [97] or removed by a cysteine wash step during the protein A chromatography step [98]. Trisulfide bond increases mAb molecular weight by 32 Da. A mAb variant with a trisulfide bond appeared to be more acidic compared to mAb with the typical disulfide bond pattern [94]. The presence of a trisulfide bond has no impact on antigen binding [95,98] or thermal stability [94].

The cysteine residues located in the heavy chains that are involved in the formation of the light and heavy chain disulfide bonds were also found to exist in the D form [99]. A detailed study showed that racemization occurred in both the heavy and light chain cysteine residues in IgG1 lambda, but only the heavy chain in IgG1 kappa in both mAbs and human natural IgG1 [100]. The level of cysteine racemization is much lower in IgG2 [100]. As both thioether and racemization are catalyzed by basic condition and involve the same disulfide bonds, a general base-catalyzed mechanism was proposed, where beta elimination of the disulfide bond results in the formation of a dehydroalanine residue and the dehydroalanine residue can either form a thioether bond or revert to the disulfide bond, where chirality is regained to result in a mixture of D- and L- cysteine residues [100]. 

Most of the modifications identified in mAbs, including free cysteine [67,101,102], alternative disulfide bond linkage for IgG4 [82,84] and IgG2 [88], thioether [93], trisulfide bond [93,95,96], and D-Cys from racemization [100] have also been reported in natural human IgGs. However, cysteinylation and the presence of incomplete disulfide bonds have not been reported in natural human IgGs. Given all the negative impacts of cysteinylation, this modification may have been eliminated from natural IgG during evolution. The same could be true for the presence of a single pair of incomplete disulfide bonds.

## 8. Glycosylation

Similar to natural IgG molecules, mAbs are N-glycosylated at the conserved Asn residues in the CH2 domain. In addition, mAbs may have N-linked oligosaccharides in the Fab region [103,104,105]. Heterogeneity related to these oligosaccharides arises mainly from galactosylation, fucosylation, and sialylation of the biantennary complex oligosaccharides. The presence of low abundance oligosaccharides, such as high mannose (Man), hybrid and bisecting oligosaccharides adds further heterogeneity to mAbs.

The three major glycoforms are the core-fucosylated structures with either zero (G0F), one (G1F) or two (G2F) galactose [104,106,107,108,109,110,111]. Galactose adds an additional mass of 162 Da. However, galactosylation has not been reported to cause mAb heterogeneity in charge or hydrophobicity. The slight separation of mAb variants with different levels of galactosylation is probably caused by conformational differences since galactose should not change the charge properties [77]. Galactosylation can cause subtle conformational changes around the glycosylation site [54,112,113,114,115]. Conflicting results have been reported regarding biological functions, but it is generally agreed that galactose might slightly impact CDC, but not antibody-dependent cellular cytotoxicity (ADCC) [106,113,114,115,116,117,118,119,120,121,122]. Galactosylation has no impact on mAb stability [113,114,123,124], nor half-life [103,117,125,126,127,128]. 

Because the absence of the core-fucose can result in enhanced ADCC [122,129], the level of core-fucose has attracted great attention for the development of mAb therapeutics, especially to establish comparability or biosimilarity. The attachment of fucose adds a mass of 146 Da. Besides mass heterogeneity, fucose has not been reported to have an impact on charge and hydrophobicity. The addition of a fucose only has a subtle impact on mAb structure [54,130,131]. For mAbs without the core-fucose, animal studies on half-life have shown conflicting results [132,133]. However, the half-life was found to be as expected in human studies [125,127]. The level of core-fucose needs to be evaluated based on the target and therapeutic goal to balance the risk versus benefit [134].

Besides the complex oligosaccharides, high mannose oligosaccharides have been commonly observed in mAbs [108,109,135]. In addition to mass heterogeneity, mAbs with high mannose oligosaccharides demonstrate a slightly different chromatographic separation when using Protein A or Protein G columns [136]. High mannose oligosaccharides cause a subtle conformational change and increase the flexibility of the CH2 domain [137,138]. Although high mannose decreased the thermal stability of mAbs, it had no impact on long-term stability [131], or aggregation propensity under accelerated conditions [124]. High mannose shows increased Fc gamma receptor binding and ADCC, due to the absence of core-fucose [139]. IgG with high mannose showed reduced activities mediated by the first subcomponent of the C1 complex (C1q) binding [140,141]. High mannose oligosaccharides with greater than five mannose residues are rapidly converted into a structure with only 5 mannose residues (Man5) in human circulation [125]. MAbs with high mannose are cleared at a faster rate compared to those with complex oligosaccharides in animals and humans [126,127,139,140,142]. 

Sialic acid and alpha 1,3-galactose are two low abundant oligosaccharides that require special attention primarily because of safety concerns. In general, the level of sialic acid of mAbs that are associated with the conserved Fc glycosylation site is low [103,109,135]. However, substantial amounts of sialic acid have been found in mAbs containing a Fab glycosylation site [103,135]. Sialic acid adds mass heterogeneity and generates acidic species [2,13,27], but does not impact antigen binding [2,13,117,119,143,144] and clearance [13,125,128]. Sialic acid has been shown to cause subtle conformational changes that are local to the glycosylation sites [137,138,145,146,147]. Studies have demonstrated that sialic acid exerts no or a negative impact on ADCC and CDC [117,119,143,144]. Among the two types of sialic acids, N-Acetylneuraminic acid (NANA) and N-Glycolylneuraminic acid (NGNA), the latter, which is commonly found in mAbs from murine cell lines [2,103,104,108], has been linked to immunogenicity [148]. Similar to sialic acid, mAbs expressed in murine cell lines may contain low levels of alpha 1,3 galactose when associated with the Fc [3,103,104,108,109,149] and at relatively higher levels for mAbs containing Fab glycosylation [135]. Alpha1,3 galactose is also considered immunogenic [150] when associated with Fab [151]. 

Several other types of oligosaccharides including hybrid, bisecting, and smaller structures, such as those lacking outer arm N-acetylglucosamine (GlcNAc) residues, are present in mAbs at extremely low levels. Hybrid, bisecting, and smaller oligosaccharides could cause a subtle conformational change [138] and have a minimal impact on ADCC [118,122,137,152] and clearance [125,126]. Because of their extremely low levels, these types of oligosaccharides are not expected to have a substantial impact on mAb therapeutic development from the safety and efficacy point of view.

The absence of oligosaccharides also contributes to mAb heterogeneity, though, at low levels [153,154,155,156]. MAbs lacking oligosaccharides showed significant conformational changes [157,158], decreased thermal stability [112,113,131,159,160], and increased aggregation propensity [123,161]. The absence of oligosaccharides has a substantial impact on ADCC and CDC [113,119,157]. Initially, animal studies showed that the absence of oligosaccharides either caused a faster clearance [157,162,163] or had no impact on half-life [127,157,160,164,165]. However, later human trials demonstrated that aglycosylated mAbs had a normal half-life [164].

Human IgG contains similar major oligosaccharide structures but higher structural diversity [108,109,166]. The levels of bisecting and sialic acid are higher in natural human IgGs compared to mAbs [108,109,166], while, high mannose oligosaccharides in natural human IgG are extremely low at approximately 0.1% [109,167]. Human IgGs have also been shown to have less than 0.2% aglycosylation [167]. NGNA and alpha 1,3 galactose, are absent from natural human IgGs [108,109,166,168]. 

## 9. Glycation

Glycation is a non-enzymatic reaction between reducing sugars and the primary amine of the lysine (Lys) side chain or the N-terminus of the light chain or heavy chain [169,170,171]. Glycation mainly occurs during cell culture as sugars are used as nutrients [169], and, to a lesser degree, during storage or accelerated conditions due to decomposition of the non-reducing sugars used in formulation [172,173]. A slightly increased level of glycation has been reported during the course of administration when a diluent containing sugars is used [174]. Glycation increases mAb molecular weight by 162 Da with each site of glycation and generates acidic species due to loss of positive charges of Lys side chains or N-termini [13,27,43,169,171]. Glycation also increases the aggregation propensity under accelerated condition [173]. Glycation in the CDRs has not been shown to decrease antigen binding [13,169,171], and even a substantial level of glycation does not affect Fc gamma, FcRn, and protein A binding [175]. Advanced glycation end products (AGEs) contribute to product coloration [176]. The presence of glycation does not impact PK in rats [13]. Glycation of mAbs continues to occur in circulation in humans at a rate that can be predicted via in vitro incubation under physiological conditions [175]. 

As expected, glycation has been detected in endogenous human IgG [175], further supporting the simple reaction mechanism between circulating IgGs and sugars in vivo.

## 10. C-Terminal Modifications

Mostly, mAbs are synthesized with the heavy chain C-terminal Lys, which can be removed during cell culture due to carboxypeptidase activity [177]. Incomplete removal results in mAbs with either zero, one or two C-terminal lysine at various levels [2,3,12,178,179]. When analyzed by mass spectrometry, heterogeneity caused by C-terminal lysine is reflected by peaks that differ in molecular weight by 128 Da. C-terminal Lys is a common cause of the generation of basic species [1,9,12,43,178,179,180]. The presence of C-terminal Lys results in the formation of less hydrophobic mAb variants [35,45]. C-terminal Lys does not impact mAb structure, stability, or biological functions including PK [2,9,13,180,181,182], though, one study demonstrated that the removal of C-terminal Lys is required for optimal CDC [183]. Interestingly, inclusion of the C-terminal Lys codon may impact mAb titer of cell culture [184]. C-terminal Lys can be rapidly removed from mAbs during circulation with a half-life of 62 minutes [185]. 

C-terminal amidation was first discovered in a recombinant monoclonal IgG1 antibody [186]. Later, it was found that C-terminal amidation is as common as C-terminal Lys removal [187]. C-terminal amidation is catalyzed by peptidylglycine alpha-amidating monooxygenase (PAM) [187]. The level of C-terminal amidation can be modulated by changing the copper concentration in the cell culture media [188] or via genetic engineering to reduce PAM activity [189]. Compared to mAbs without C-terminal Lys, a loss of glycine and conversion of the newly exposed amino acid carboxyl group to an amide group results in a net molecular weight decrease of 58 Da. MAbs with C-terminal amidation are separated as basic species [43,186,188]. MAb variants without C-terminal Lys or without both Lys and Gly showed no difference in structure, stability, function, and PK [181]. 

The overall level of C-terminal amidation in natural human IgG is extremely low, approximately 0.02% or lower [185,187]. 

## 11. Uncommon Modifications

Several of the reported modifications only contribute to mAb heterogeneity at very low levels or in only a limited number of cases. 

Low level of sequence variation has been observed for several mAbs [190,191,192,193,194,195,196,197,198], which is expected to be the norm rather than the exception because of the inherent errors in protein transcription and translation. An mAb variant with the heavy chains containing amino acids that were coded by part of the intron sequence was also found [3]. Recombination between light chain and heavy chain sequences has been reported to result in a minor mAb species where the heavy chain containing a portion of the light chain sequence [199]. 

Aside from the few cases of amino acid variation, several rare chemical modifications can occur at various stages. Methylglyoxal generated during cell culture has been shown to modify an mAb at arginine (Arg) residues, resulting in molecular weight increases by 54 Da or 72 Da and generation of acidic species [200]. Metals can catalyze oxidative carbonylation of several surface-exposed residues including Arg, Proline (Pro), Lys, and Thr [201]. When exposed to light, histidine (His) can be oxidized [202], which can further lead to His–His cross-linking [203]. Cysteinylation, which frequently occurs at non-canonical cysteine residues, has also been reported at canonical cysteine residues in IgG2 [12,96] and is probably due to the relative instability of the IgG2 disulfide bond linkage around the hinge region. The presence of tyrosine sulfation resulted in the formation of a distinct acidic peak for a mAb expressed in Chinese hamster ovary (CHO) cells [204]. Modification of light chain and heavy chain N-termini by maleuric acid has been detected in a mAb expressed in transgenic goats [205]. During storage, the N-terminal primary amine or lysine side chain of mAbs can be modified by citric acid or its degradation products [206,207]. In addition to glycosylation of the conserved Asn residues in the Fc region or glycosylation of Asn in the consensus sequence in the variable domains, O-fucosylation of a serine residue in the light chain CDR1 [208] and N-glycosylation of Asn in non-consensus sequence and Gln [209,210] have also been reported.

MAbs expressed in mammalian cell lines have been extensively characterized. However, novel modifications are expected whenever new cell culture media or formulations are used. The use of alternative expression systems is also expected to lead to novel modifications that are specific to the selected organism. Novel and non-clinically qualified modifications naturally bear higher safety risks, and, thus, requires thorough evaluation.

## 12. Heterogeneity in the Broader Scheme

### 12.1. Stability

ICH Q6B states that “degradation of drug substance and drug product, which may occur during storage, should be considered when establishing specifications.” ICH Q6B also discusses the concept of “Release limits vs shelf-life limits”, where tighter release limits will ensure that product at the end of shelf life can meet the acceptance criteria to maintain safety and efficacy. 

Regarding stability, the aforementioned PTMs can be classified into two categories. The first category includes modifications that are catalyzed by enzymatic reactions. Those modifications include signal peptides, various glycoforms, C-terminal Lys removal, and C-terminal amidation. These types of modifications are not expected to continue during storage because of the lack of their respective enzymes in the drug substance and drug product. However, the levels of these modifications can potentially impact other degradation pathways, and, thus, stability. For example, the subtle conformational difference in mAbs with various oligosaccharides and the substantial conformational difference caused by the lack of oligosaccharides are expected, at least in theory, to impact other modifications by modulating surface exposure and inter-molecule interactions. The second category includes modifications that are dependent only on environmental factors, such as pH, temperature, and light exposure. Modifications in this category include N-terminal Gln and Glu cyclization, deamidation, isomerization, succinimide intermediate formation, oxidation, cysteine and disulfide bond related modifications, and glycation. Modifications in this category are expected to continue to occur during storage.

Overall, PTMs are, either indirectly or directly, linked to mAb stability. Detailed characterization of drug substances at the time of lot release and understanding of the degradation pathways derived from forced degradation, and stability studies can ensure mAb stability during shelf-life for consistent safety and efficacy.

### 12.2. Comparability and Biosimilarity

Comparability is required when process changes are introduced, which is inevitable during development. Q5E states that “The demonstration of comparability does not necessarily mean that the quality attributes of the pre-change and post-change product are identical, but that they are highly similar and that the existing knowledge is sufficiently predictive to ensure that any differences in quality attributes have no adverse impact upon safety or efficacy of the drug product”. Scientific understanding of the chemical nature of PTMs and their impact on safety and efficacy is critical to establishing comparability, especially when a quality attribute is outside of the historical range. 

MAb heterogeneity is also central to the development of biosimilar products. Given the requirement that the primary sequence of the originator and a biosimilar product should be identical, it becomes clear that similarity is mainly dependent on various PTMs. 

In-depth characterization of mAb heterogeneity plays an essential role in establishing comparability and biosimilarity. The National Institute of Standards and Technology mAb (NISTmAb) tryptic peptide spectral library can be used as a good reference for those detailed comparisons [211], as it contains an extensive list of modifications, including the commonly observed analytical artifacts, which should be differentiated from true modifications.

### 12.3. Antibody-Drug Conjugate

Antibody-drug conjugates (ADCs) take advantage of the specificities of mAbs to deliver functional molecules to targets, and commonly, high toxicity compounds, to cancer cells [212]. MAb heterogeneity, thus, becomes an integral characteristic of ADCs, and exerts similar impact on structure, and stability. The microenvironment of the conjugation sites including solvent accessibility and charges has been demonstrated to have a substantial impact on the in vivo stability and activity of ADCs [213]. The presence of trisulfide bonds, for example, has also been shown to affect conjugation and the resulting drug-to-antibody ratio (DAR) [16,214]. Higher levels of heterogeneity have been reported for ADCs based on IgG2 mAbs, which are known for their various disulfide bond isoforms and difference in disulfide bond accessibility [215]. 

## 13. Conclusions

Heterogeneity is recognized as a common feature of mAbs due to modifications that cause IgG variants or proteoforms that differ in molecular weight, charge or hydrophobicity. MAb variants are required to be evaluated to establish their structure–function and safety relationships. In addition, different variants may have (or not) different impacts on stability, which is ultimately linked to safety and efficacy.

A wealth of information has been accumulated over the past decades. Such knowledge can be generally used to define the quality target product profile and applied to the assessment of developability of clinical candidates during the early phase of pharmaceutical development. Later in development, molecule-specific modifications are observed and managed throughout the lifecycle of the selected mAb.

## Figures and Tables

**Table 1:** Micro-heterogeneity natural IgGs and recombinant mAbs.

Modifications	Natural	Recombinant	Resulting Heterogeneity
N-terminal modifications			
PyroGlu	100% pyroGlu	Varied levels	Mass, charge for Gln to pyroGlu
Truncation	Not expected	Rare and low	Mass
Signal peptides	Not expected	Low	Mass and charge
Asn deamidation	Substantial level	Common, varied levels	Mass and charge
Asp isomerization	Not expected	Common, varied levels	Charge and hydrophobicity
Succinimide	Not expected	Common, varied levels	Mass, charge, and hydrophobicity
Oxidation	Low	Met, Trp, Cys, His	Mass and hydrophobicity
Cysteine related modifications			
Free cysteine	Low	Low	Mass, charge and hydrophobicity
Alternative disulfide bond linkage	Common	Common	Charge
Trisulfide bond	Extremely low	Low	Mass and charge
Thioether	Low	Low	Mass
Glycosylation	Common	Common	Mass and charge
Glycation	Common	Common	Mass and charge
C-terminal modifications			
C-terminal Lys	Complete removal	Common, varied levels	Mass, charge and hydrophobicity
C-terminal modifications	Not detected	Low varied levels	Mass and charge

## Abbreviations

ADC	Antibody-drug conjugate
ADCC	Antibody-dependent cellular cytotoxicity
AGE	Advanced glycation end product
Asn	Asparagine
Asp	Aspartate
Arg	Arginine
C1q	First subcomponent of the C1 complex
CDC	Complement-dependent cytotoxicity
CDR	Complementarity determining region
CE-SDS	Capillary electrophoresis sodium dodecyl sulfate
CH2	Heavy chain constant domain 2
CHO	Chinese hamster ovary
CQA	Critical quality attribute
Cys	Cysteine
Da	Dalton
Fab	Fragment antigen binding
Fc	Fragment crystallizable
FcRn	Neonatal Fc receptor
GlcNAc	N-acetylglucosamine
Gln	Glutamine
Glu	Glutamate
Gly	Glycine
His	Histidine
ICH	International Conference on Harmonization
IgG	Immunoglobulin G
IsoAsp	Isoaspartate
LC-MS	Liquid chromatography mass spectrometry
Lys	Lysine
mAb	Monoclonal antibody
Man	Mannose
Met	Methionine
NANA	N-Acetylneuraminic acid
NGNA	N-Glycolylneuraminic acid
PAM	Peptidylglycine alpha-amidating monooxygenase
PK	Pharmacokinetics
Pro	Proline
PTM	Posttranslational modification
PyroGlu	Pyroglutamate
QTPP	Quality target product profile
SDS-PAGE	Sodium dodecyl sulfate polyacrylamide gel electrophoresis
Thr	Threonine
Trp	Tryptophan
